# The Need for Review and Understanding of SELDI/MALDI Mass Spectroscopy Data Prior to Analysis

**Published:** 2007-02-19

**Authors:** William E. Grizzle, O. John Semmes, William Bigbee, Liu Zhu, Gunjan Malik, Denise K Oelschlager, Barkha Manne, Upender Manne

**Affiliations:** a University of Alabama at Birmingham, Birmingham, AL;; b Eastern Virginia Medical School, Norfolk, VA; c University of Pittsburgh Cancer Institute, Pittsburgh, PA, USA

**Keywords:** bias, specimens, specimen processing, mass spectrometry, serum, cancer detection

## Abstract

Multiple studies have reported that surface enhanced laser desorption/ionization time of flight mass spectroscopy (SELDI-TOF-MS) is useful in the early detection of disease based on the analysis of bodily fluids. Use of any multiplex mass spectroscopy based approach as in the analysis of bodily fluids to detect disease must be analyzed with great care due to the susceptibility of multiplex and mass spectroscopy methods to biases introduced via experimental design, patient samples, and/or methodology. Specific biases include those related to experimental design, patients, samples, protein chips, chip reader and spectral analysis. Contributions to biases based on patients include demographics (e.g., age, race, ethnicity, sex), homeostasis (e.g., fasting, medications, stress, time of sampling), and site of analysis (hospital, clinic, other). Biases in samples include conditions of sampling (type of sample container, time of processing, time to storage), conditions of storage, (time and temperature of storage), and prior sample manipulation (freeze thaw cycles). Also, there are many potential biases in methodology which can be avoided by careful experimental design including ensuring that cases and controls are analyzed randomly. All the above forms of biases affect any system based on analyzing multiple analytes and especially all mass spectroscopy based methods, not just SELDI-TOF-MS. Also, all current mass spectroscopy systems have relatively low sensitivity compared with immunoassays (e.g., ELISA). There are several problems which may be unique to the SELDI-TOF-MS system marketed by Ciphergen^®^. Of these, the most important is a relatively low resolution (±0.2%) of the bundled mass spectrometer which may cause problems with analysis of data. Foremost, this low resolution results in difficulties in determining what constitutes a “peak” if a peak matching approach is used in analysis. Also, once peaks are selected, the peaks may represent multiple proteins. In addition, because peaks may vary slightly in location due to instrumental drift, long term identification of the same peaks may prove to be a challenge. Finally, the Ciphergen^®^ system has some “noise” of the baseline which results from the accumulation of charge in the detector system. Thus, we must be very aware of the factors that may affect the use of proteomics in the early detection of disease, in determining aggressive subsets of cancers, in risk assessment and in monitoring the effectiveness of novel therapies.

## Introduction

Surface enhanced laser desorption/ionization time of flight mass spectroscopy (SELDI-TOF-MS) is a relatively new high throughput proteomic technique that has been reported to be useful in the early detection of disease. Specifically, SELDI-TOF-MS has been used to analyze samples of body fluids to aid in the early detection of multiple neoplastic processes. Serum has been the major bodily fluid utilized in most studies reported to date (Table 1).

Before any attempt is made at analysis of data of any form, the statistician/bioinformaticist should be thoroughly familiar with the source and accuracy of the data. The obvious trite statement applies: junk to the statistician equals junk from the statistician; thus, care should be taken to understand the quality of the data prior to analysis. The purpose of this manuscript is to alert those analyzing SELDI-TOF-MS, other mass spectroscopy techniques, and other proteomic data of the potential sources of incorrect, inaccurate and/or biased data. The sources of problematic data can be subdivided into the following: experimental design, patient, sample, protein chip, chip reader, measures of the spectrum including peak identification, peak comparisons, and algorithms of spectral analysis.

## Experimental Design

The importance of careful experimental design involves each of the potential sources of biases listed above. For example, great care must be taken in identifying the patients to be studied and especially in carefully choosing control patients. For example, how does one ensure that controls do not have the subclinical form of the disease being studied? As part of the experimental design, the type of sample to be analyzed needs to be considered; for example, would serum, plasma, urine, saliva, cytological specimens, or combinations of these be the best samples to study.

Also, samples from cases and controls must be collected, processed and stored consistently. The use of SELDI-TOF-MS in early detection usually requires processing of samples using robotics, performing the assays in triplicate, and consistency in processing (e.g., dilution) of the sample before adding the triplicate of the sample to the chip. The type of chip(s) should be selected before the experiment (Table 2) and enough chips from one lot should be assembled to complete the experiment if practicable within the shelf lives of chips. Samples should be applied to the chip in a manner selected to prevent bias and errors introduced from the methodology. Thus, because random and consistent errors may arise due to spot, chip, and day of assay, error can be minimized by ensuring that each of the triplicates is placed on a different spot (e.g., not more than one of a triplicate on spot A), each on a different chip (i.e., no more than one of the triplicates on the same chip) and each of the triplicates should be analyzed on a different day. Given these restrictions, cases and controls should then be applied randomly and blindly to the chip based upon statistical considerations. Thus, each of the triplicates is analyzed within one of three groups. Each of these groups should be analyzed on separate days. Spots and chips are randomly assigned except that spots and chips must be different on each of the triplicates of one specimen (see Table 3).

**Figure 1:**
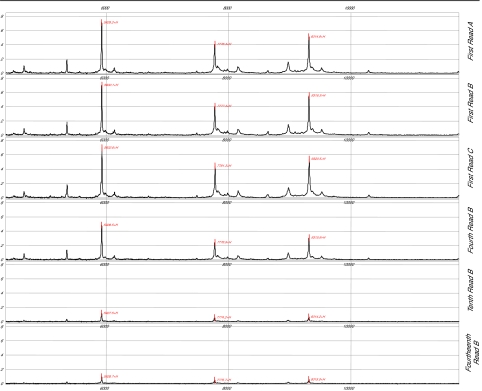
Figure 1 demonstrates the spectral decreases upon multiple laser shots in same area. The same sample was applied to spots A, B, and C and these spots were analyzed initially with 5 laser shots per spot to demonstrate consistency of spectral pattern. Following 3 additional samplings of spot B, at 5 laser shots each at the same site, the decrease in the spectrum is clear (4^th^ 5 shot read of spot B). After a total of 10 and 14 5 shot samplings at the same site on spot B, a marked decline in sample intensity was noted (10^th^ read of spot B and 14th read of spot B respectively).

Similarly, a consistent aliquot of a standard control sample should be used (at least one per chip) and the standard control sample also should be loaded on chips without bias as to the spot on the chip to which it is loaded. The chip should be prepared using a robotic system within one day of analysis. The energy absorbing molecule (EAM) should be chosen with care to match the molecular weight range of interest. If a whole spectrum is of interest 1000 Daltons to 200,000 Daltons, then multiple runs of a sample will be necessary, each run over a specific molecular weight range with a separate set of triplicates and different adjustments including to laser and detector. For each range of interest (e.g., 1 kD to 15 kD) an EAM should be selected and the machine should be calibrated for this spectral area. EAMs vary in efficiency based upon the molecular weight range being analyzed. Thus, an area such as from 50 kD to 100 kD will require a different EAM and a specific set of molecular weight standards (e.g., 5 to 7 purified proteins) which bridge these molecular weights. The points at which the laser samples the spots, should be selected so as to not exhaust the sample-EAM matrix (Figure 1). If a directed (e.g., peak identification) approach to analysis is to be used, a careful, consistent method of peak identification should be utilized and the alignment of peaks should be consistent. The instrument should be calibrated periodically and the consistency of the instrumental output should be verified using the standard samples as part of the quality control program. Finally, there must be a consistent approach to the analysis of data.

## Patient and Controls

The general approach of proteomics is to compare one condition with another; thus, in studies involving the early detection of a disease, patients with the disease (cases) are compared with patients without the disease (controls). The first very important issue is to identify what are the clinical (research) and other parameters that define a good control. For example, controls for prostate cancer (PCa) should not have prostate cancer but should be males who have conditions such as benign prostatic hyperplasia (BPH) which may mimic PCa as well as be within the age range that PCa usually occurs > 50 years of age. Familial cases may need separate controls. The usual normal range for PSA is ≤ 4 ng/ml; however, based upon the Prostate Cancer Prevention Trial (PCPT) about 20% of patients with PSA values ≤ 4 ng/ml, have PCa (Thompson et al, 2004). Similarly, about 60% of patients with PSA values from 4 to10 ng/ml have no PCa but rather BPH. Men with PSA values much greater than 100 ng/ml are more likely to have PCa (Urban et al, 1999). Although no control group is perfect, one might select controls for a serum based study of PCa as being males with PSA ≤ 10 ng/ml, a normal digital rectal examination, normal prostatic ultrasonography, and a recent negative biopsy (at least sextant) of the prostate taken **after** the sample of serum was obtained because biopsy of the prostate may “activate” the prostate for several weeks subsequently (Urban et al, 1999). Controls above 4 ng/ml but less than 10 ng/ml are chosen because most samples in this range do not have PCa and controls should not exclude proteomic changes secondary to benign prostatic hyperplasia (BPH) (Grizzle et al, 2003, 2005). Such controls might be collected prospectively or their samples might exist in a tissue bank; however, it is critical that all conditions of cases (site, collection, storage, etc.) match controls. Because various metabolic states may affect proteomic assays (e.g., diurnal rhythm, chronic diseases, stress), the metabolic and other conditions under which the control samples were collected should mirror the conditions under which the samples from patients with PCa were collected. For example, either a group of males all of whom had rheumatoid arthritis or a group of males all of whom had undergone a glucose challenge 2 hours prior to obtaining samples would be a bad control group for a group of males with PCa and the normal incidence of rheumatoid arthritis and a fasting state prior to sampling. It is necessary that such conditions average out and thus their contributions to the analysis of spectral patterns would be noise. The patterns of eating of disease groups can result in an important bias because some medical facilities may require patients to be fasting when visiting a clinic/hospital while other locations may not. Identifying members of the control groups for other cancers is just as demanding. Note that the age, sex and health of the control group should match the case group. One way to approach this is to require the same number of controls as cases be collected from each site (i.e., if 30 cases of PCa are collected from site A, then site A would also supply 30 controls).

Because some of the spectral peaks in SELDI-TOF-MS analysis have been attributed to nonspecific inflammatory peaks, in some studies of neoplasia it would be useful to have additional separate groups of patients without the cancer being studied to identify nonspecific proteins which may be associated with conditions such as rheumatoid arthritis, systemic lupus erythematosis, sepsis, and other tumors. Such groups should not be used in training the algorithm but rather to test the algorithm as to the specificity of the informative peaks selected by the algorithm.

Specific issues to consider in the selection of members of the case group would be the type(s) of cancers studied. If the focus were to be on early detection of PCa, one would not want to evaluate samples from males who had metastatic PCa at the time of sampling. Similarly, Gleason scores might be chosen to separate more indolent PCa (Gleason ≤ 6) from more aggressive (Gleason ≥ 7) PCa. Ultimately in early detection, analysis of samples of serum obtained one to several years prior to the diagnosis of a specific cancer is necessary (Pepe et al., 2001).

When the cancers of an organ such as lung are of various types, e.g., squamous cell carcinoma, adenocarcinoma, bronchoalveolar, small cell (oat cell) un-differentiated and “other”, then each type of cancer should be evaluated separately. Although it is possible that all types of carcinomas of the lung could be separated from controls by SELDI-TOF-MS analysis, more specific peaks will probably be identified if each type of tumor were analyzed as a separate group. For such cases, each type of tumor should utilize a different case group although the control group could remain the same, except for bronchoalveolar carcinoma which usually is not associated with smoking as a risk factor. Also, a separate algorithm should be developed and optimized for each type of tumor.

**Figure 2:**
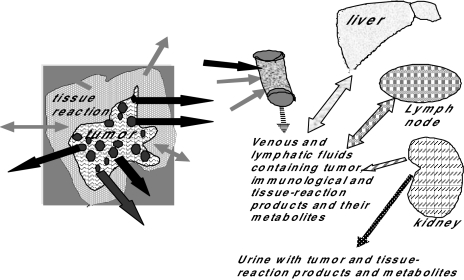
Figure 2 demonstrates that molecular markers in bodily fluids that may be used in the diagnosis of cancers may come from multiple sources. The tumor itself may produce markers such as CEA (colorectal cancer) that are produced by the tumor. Other tumor products may circulate and induce changes in distant tissues (e.g. liver and kidney) affecting the synthesis or metabolism of specific molecules. The stromal and inflammatory response to the tumor may also modulate proteins (e.g, cytokines) in serum.

**Figure 3:**
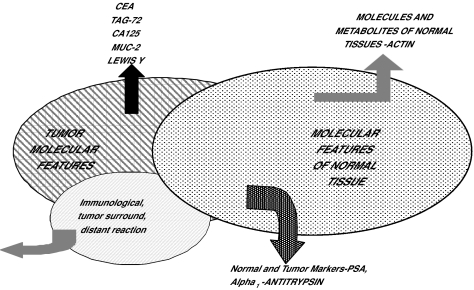
Figure 3 emphasizes that some markers such as oncofetal tumor molecules are produced directly by the tumor while other tissue specific molecules such as PSA may be produced by the uninvolved tissues in addition to the tumor. Patterns of all proteins in bodily fluids depend upon multiple factors including where the contents of dying cells are dumped as well as the rate of cellular death.

When an individual has cancer, multiple changes may occur in the spectral patterns of proteins in blood. As suggested in Figures 2 and 3, these changes include spectral peaks that correlate with molecules that are released from the tumor and molecules that are produced by local and distant responses to the tumor. Similarly, regulatory molecules may be released from the tumor and/or the epi-reaction and may modulate the production of proteins by distant tissues (e.g., acute phase reactants produced by the liver). The immune system also will produce reactions to the products of the tumor including cellular contents released into the circulation Other molecular changes may be modulated by tumors including the rate at which specific molecules are excreted or metabolized by the kidney or biliary-colorectal system. Also, specific proteolytic enzymes may be produced by a tumor or surrounding cells and these enzymes may affect patterns of proteins in serum and other bodily fluids. Ultimately, carrier proteins may bind smaller molecules; this may affect their analysis, for example, by concentrating them (Grizzle et al, 2005). All these potential sources of molecular changes in the spectral proteins in serum may combine to form spectral patterns which may be characteristic of the presence of a specific disease, e.g., a specific type of tumor.

**Figure 4:**
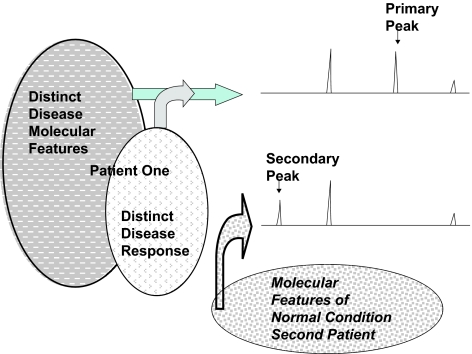
Figure 4 demonstrates two types of peaks observed in spectra when comparing cases with controls. The molecular features of the control lower spectrum demonstrates three peaks. These would usually be seen in any patient without disease. In the spectrum of the diseased patient, a new peak (primary peak) is present in the spectrum. This would probably result from a product produced because of the disease. Of interest, a peak (secondary peak) present in the spectra of most patients without disease is now absent from the spectrum of the diseased patient. Such peaks are not understood but may represent tumor-normal organ cross talk to reduce production of a protein or the production of an enzyme by the diseased state which metabolizes the protein of the secondary peak.

In spectra from cases and controls, two different types of informative peaks may be identified. One type of informative peak is highest in the cases of cancer (Figure 4). We designate such peaks as “primary” because they are suggestive of a primary product arising because a tumor is present in a patient. The second type of informative peak is lower in the cases with cancer than in control serum (Figure 4). We designate such peaks as “secondary” because their presence suggests that the presence of the cancer in a patient causes a decrease in a peak that usually is present in serum of normal individuals. This suggests the presence of the tumor increases the degredation of the secondary peak either due to increased proteolytic activity or, the production of a strong binding protein. Also, such changes in peaks may occur via the modulation of production of a molecular species and/or of excretion of molecules via kidney or biliary-colorectal system, by binding and removal via the immune system, or via a combination of these processes.

**Figure 5:**
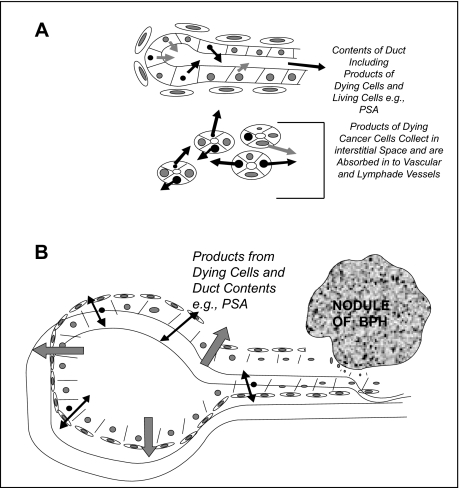
Figure 5A is a cartoon which suggests how the secretions of living cells and the contents of dying cells of normal prostatic glands may exit the body without being absorbed into the vascular system. In contrast, dying cells of prostate cancer dump their contents into the interstitutium and these products are likely to be absorbed into the vascular system. Figure 5B demonstrates that as benign prostatic hyperplasia develops that glandular contents may be blocked from the usual pathway. Subsequently the glands may become dilated and/or inflamed and contents including PSA may leak from the lumen of the gland into the interstitial space.

Sometimes it is not the concentration of a molecular species that is produced by a tumor or that is present in the cells of tumors, but rather where the contents of tumor cells are released and the rate at which tumor cells die that control the levels of a protein in fluids. For example, the cells of adenocarcinoma of the prostate (PCa), in general, have a lower concentration of prostatic specific antigen (PSA) than the cells of normal prostatic tissues. How then can PSA be a sensitive marker of the early detection of PCa? First, when the cells of normal prostatic tissues die, they dump their contents into the lumina of the normal prostate glands and the contents of the glands are ultimately cleared in the ejaculate (Figure 5A). In contrast, when the cells of PCa die, they are not located in an intact ductal system and thus these cells dump their contents into the interstitial space so that their contents are absorbed by the vascular-lymphatic system (Figure 5A). Similarly, if benign ducts become blocked by changes of BPH or by the accumulation of concretions, their wall may leak products such as PSA into the interstitial space (Figure 5B).

### Samples

The choice of the best type of sample is controversial. Some investigators use plasma to avoid the activation of the coagulation system and consequent release of factors from platelets and proteins associated with coagulation. Others prefer serum in which coagulation has removed some high concentration proteins.

**Figure 6:**
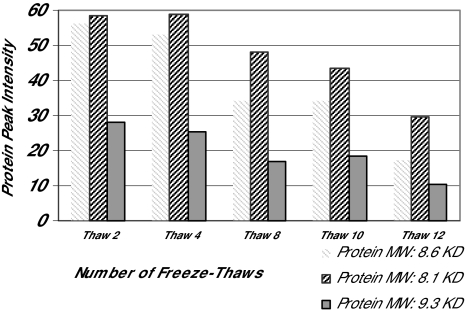
The bar graph of figure 6 demonstrates the decline in the peaks of three unidentified proteins in serum that follow multiple freeze thaw cycles of samples of serum. The pattern of decline varies with specific peaks; however, most peaks do not decline greatly until after at least three freeze thaw cycles.

**Figure 7:**
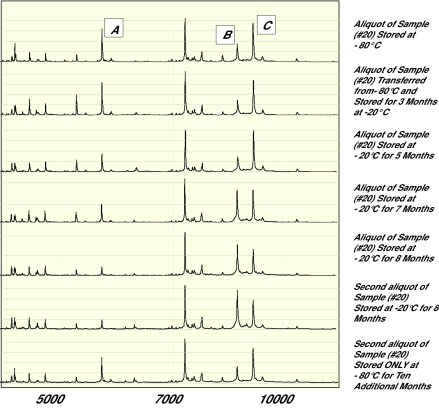
Figure 7 demonstrates that storage of aliquots of a sample at −20°C (non-self defrost) for more than 6 months results in changes in peak amplitudes (A) and peak amplitude ratios (B vs C). Such changes were not noted on storage of an aliquot from the same original specimen for 10 months at −80°C.

Once the type of sample is chosen, the sampling conditions need to be standardized. The choice of sample collection and storage containers may influence spectral results. While glass sample collection tubes may activate specific proteins, plastic collection and storage containers may contaminate specimens with plastic components. Because plastics usually are poly-molecular forms (e.g., polyvinyl chloride), plastic contaminants may present as repeating spectral peaks, each separated by a standard molecular weight, usually about 100 to 200 Daltons. Similarly, additives to tubes (e.g., anticoagulants such as heparin) may influence spectral patterns. A minimum sample size should be selected and samples should be aliquoted to minimum sizes shortly after collection to avoid repeated freeze-thaw cycles. As demonstrated in Figure 6, peak amplitudes may decline upon several freeze-thaw cycles. The conditions between the collection of samples and storage of specimens should be standardized. It also is important to have conditions of storage consistent; for example, if cases are to be collected in the next two years, one does not want to use controls from an archival serum collection stored for 10 years or more at −70°C. It is critical that samples be stored at best at −70°C or colder. Storing samples at −20°C or warmer, even in a non-self defrost freezer, may result in degredation of proteins as measured by spectral changes (Figure 7). We are aware of MALDI-TOF-MS data that indicate spectral peaks do not change upon storage at −70°C or colder over a 4 year period.

The proteomic systems may be very sensitive to small errors in pipetting and thus robotic processing of samples including the addition of EAM to sampling spots is recommended. In some cases the protocol may call for removal of proteins normally present at high concentrations (e.g. albumin, immunoglobulins). The binding of proteins/peptides to carrier proteins (e.g., albumin) may act to concentrate low molecular weight proteins normally expected to be present in low concentrations or to be of small size which can be cleared rapidly by renal excretion (Grizzle et al, 2005). One must be aware that the method of removal of carrier proteins also may remove small molecules at low concentrations that are carried by, for example, albumin. Also, whether or not samples are to be diluted must be considered. It should be noted that the results may be sensitive to the extent of dilution so various dilutions should be tested on aliquots of the same sample. Foremost, it is critical that cases and controls be collected, processed and stored under the same general conditions.

#### Protein Chip

The SELDI system is designed for relatively high throughput analysis by chromatographic separation of specific categories of molecules from complex molecular mixtures such as serum and for efficiently analyzing these molecules via time of flight mass spectroscopy. This task is performed using “protein chips” which are metal chips with usually 8 sample spots. Each spot is an area of the metal chip to which specific chromatographic material is strongly attached. When complex samples are applied to a spot, molecules with specific biochemical properties are chromatographically bound by the spot and are retained on the spot even after the spot is washed extensively to remove unbound material. The chips currently available from Ciphergen are listed in Table 2. For a more detailed explanation of how SELDI-TOF-MS operates see references 2, 12 and 19.

It is important to understand that each type of chip (e.g. IMAC3 - copper activated) will produce a different protein spectra from the same sample than that produced by other types of chips. Therefore, using the same sample of serum, data from an IMAC3 Cu chip will be different from data obtained using an H50 chip. However, when similar chips are used, IMAC3 copper activated and IMAC30 copper activated, the spectra are more likely to be similar but not necessarily identical. Although users should be aware that when Ciphergen adds the hydrophobic boundary other parameters of the binding may change such that binding to the WCX2 chip differs from binding to the CM10 chip. In analysis, data on the same peaks from different types of chips should not be combined in training sets or testing sets because one cannot be certain that identical peaks are being evaluated.

Several issues related to chip characteristics may affect the results of SELDI-TOF-MS analysis. First, proteins present in large concentrations (10,000 “X” of 10 kD protein) which have the same binding characteristics of proteins present at lower concentrations, 100 “X” of 15 kD protein may saturate binding characteristics of sample spot and prevent the retention of the protein present at a lower concentration.

Once proteins are bound to the chip and the EAM is applied, the laser may not free/ionize equivalently all the proteins that were bound originally. As previously demonstrated in Figure 1, after 20 to 30 laser shots to the same site, there is a marked deterioration in the spectral characteristics from that site.

#### Spectra

Careful calibration of the instrument with proteins of known molecular weight and binding characteristics is extremely important because the raw data are measured as times of flight of the ions and the software must convert these “times” to molecular weights via the calibration step. Before samples are run, the molecular weights of interest should be determined and the instrument should be calibrated using molecular weight standards which are appropriate for the molecular weight range of interest. As discussed, if various areas of the spectrum are of interest, multiple triplicates of the same sample should be run for each molecular weight range of interest. Thus a set of peaks in the range of 2,000 to 15,000 Daltons should be measured and analyzed separately from a set of peaks from 30,000 to 60,000 Daltons. This includes using a different EAM as well as a different set of calibration standards covering the range of 30 kD to 60 kD. At each range different laser and detector settings also may be required.

### Analysis of Spectra

Once the spectral data are obtained either a “directed” or “non-directed” approach is taken in analysis. In the “non-directed” approach, an amplitude for each mass to charge (M/Z) position in the spectra is determined and analysis begins after this determination. This approach may be more sensitive to changes in peak location than the “directed” approach in which peaks are identified and peaks are matched based upon the resolution (± 0.2% mass) of the Ciphergen instrument. Alternatively a method of analysis based on determining the area under the curve (AUC) of spectral peaks can be used (Meleth et al, 2005). Subsequently the amplitude or the area under the curve of a spectra peak is determined. For each such measurement, informative peaks and AUCs are identified that separate disease from non-disease. Before choosing the first peak for an analytical algorithm, one could make a controversial distinction among peaks classifying them either as primary or secondary and evaluating their typical amplitudes or AUC, peaks with smaller amplitudes or AUCs may be less reproducible and hence less reliable in algorithms. Also, even though it has been proposed that changes that are greater in magnitude cause secondary peaks to decrease in disease patients in comparison to increases in primary peaks, the choice for the first peak in a decision algorithm might be a primary peak with a high amplitude due to the potentially more straight forward relationship with the disease process and a greater reliability in comparisons of disease with non-disease.

How variable is the peak location (resolution) and peak amplitude when samples are run on the same day on the same machine? On different days on the same machine? On different machines? Our validation study of prostate cancer (Semmes et al, 2005) has demonstrated that SELDI-TOF-MS machines at widely separated sites can be calibrated and standardized and that separate sites can identify blindly the same diagnostic peaks that previously have been shown to be important in the diagnosis of prostate cancer (Semmes et al, 2005).

All areas of the SELDI-TOF-MS spectrum are not the same from the standpoint of the early detection of disease. Areas of molecular weight of less than 1000 are in an area that is not standardized so that molecular sizes in this region cannot be determined accurately. Also, in the spectral area of less than 2000, peaks may be secondary to components of the energy absorbing molecule and/or other contaminants from, for example, plastics and the anti-coaogulants in collection tubes. Instrumental noise may in addition be present in spectral areas less than 1000 M/Z. In the spectral area above 50,000 D, many molecular species such as albumin are present in very high concentrations. Also, in our experience, the system is not very useful in detecting proteins of 50 kD or greater without adjustments (Malyarenko et al, 2005).

Because the Ciphergen system is a low resolution mass spectrometer, a protein of molecular weight 10,000 D with a concentration of 1000 “X” will prevent a protein of 10,020 D with a concentration of 100 “X” and similar binding and release characteristics for the chip utilized from being detected.

**Figure 8:**
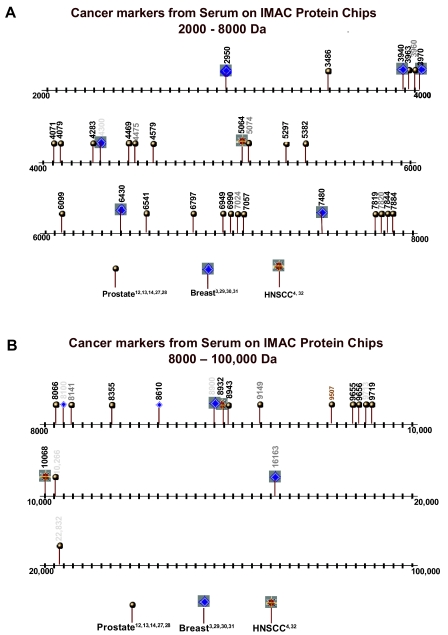
The spectra in Figure 8A (titled “Cancer markers from Serum on IMAC Protein Chips 2000 – 8000 Da”) and 8B (titled “Cancer markers from Serum on IMAC Protein Chips 8000 – 100,000 Da”) demonstrate some of the informative spectral peaks (e.g. peak locations) reported in the literature for the early detection of prostate, breast and head and neck tumors as detected using serum samples on IMAC copper activated protein chips.

**Figure 9:**
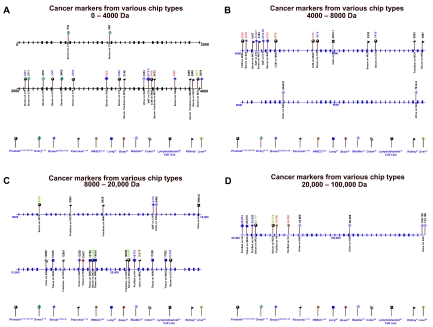
Figure 9A is depicts cancer markers from various chip types, 0 to 4000 Da. Figure 9B depicts cancer markers from various chip types 4000 to 8000 Da. Figure 9C depicts cancer markers from various chip types 8000 to 20,000 Da. Figure 9D depicts cancer markers from various chip types 20,000 to 100,000 Da. The spectra in Figure 9A–D demonstrate the informative spectral peaks (e.g., peak locations) reported in the literature (except those of Figure 8) for the detection of neoplasias in multiple organ systems using multiple samples and various types of protein chips.

Diamandis has published several criticisms of the general SELDI approach (Diamandis 2003, 2004). In each of these criticisms Diamandis has argued that for the same type of cancer, different laboratories should be identifying the same peaks. This actually should not be the case and even for prostate cancer the identification of different peaks should be the rule rather than unusual result (Grizzle and Meleth, 2004) because different studies have used different chips which bind different proteins and because 100s of peaks may separate cancer from non-cancer and this plus the algorithms chosen may result in different peaks being selected. Similarly, Diamandis (23,24) and others have argued that SELDI may be identifying peaks that are characteristic of inflammatory aspects of neoplasia or epiphenomena of cancers in general (Malik et al, 2005). This clearly is an important issue not only with SELDI-TOF-MS but also with any current forms of mass spectroscopy which have sensitivities of orders of magnitude less than the sensitivities which are necessary to detect tumor products such as PSA and CA125. Some of the peaks identified to date and their association with a specific cancer are demonstrated in Figures 8 and 9. Of interest is that peaks for various cancers have varied to date; however, based on our argument this may be serendipitous rather than an indication that the peaks are specific for a specific cancer.

Types of measurements and a discussion of issues regarding what is to be measured in spectra and various analytical approaches are discussed extensively in the other manuscripts of the volume and are beyond the scope of this manuscript. However, it is critical that all studies published include a learning/training set of samples followed by analysis of a test set of independent samples.

## Figures and Tables

**Table 1:** Summary of some of the SELDI/MALDI-TOF-MS Cancer Case/Control Serum Proteomic Profiling Studies

Organ Site	ProteinChip Type	Number of Patients in Learning/Training Set		Results of Training Set		Number of Patients in Test Set		Results of Test Set		Number of Peaks	Reference

		Controls	Cancer	Sensitivity	Specificity	Controls	Cancer	Sensitivity	Specificity		
Bladder	WCX2 weak cation exchange	104	87	87	84	18	21	78	67	7	1
Breast	IMAC3 nickel activated	66	42	N/A	N/A	66	103	85^2^	91^2^	3	2
Breast	IMAC3-Cu SAX-2	89^3^ 30^6^	45 30^6^	N/A 82 (84)^4^ 90	N/A 85 (90)^4^ 97	89^3^ 30^6^	45 30^6^	N/A 80 (78)^5^ 90^7^	N/A 79 (83)^5^ 93^7^	4 3 4	3
Head and Neck	IMAC3 copper activated	75	75	91	88	27	24	83	100	3	4
Head and Neck	MALDI	95	66	N/A	N/A	48	33	73^8^	90^8^	45	5
Kidney	WCX2 weak cation exchange	21	15	N/A	N/A	21	15	87^9^	85^9^	5	6
Liver	IMAC3-Cu WCX2	20	38	N/A	N/A	20	38	90^10^	92^10^	6 4	7
Lung	WCX2 weak cation exchange	51	30	N/A	N/A	31	15	93	97	3	8
Ovary	C16 hydrophobic interaction	50	50	100(?)	100(?)	66	50	100	95	8	9
Ovary	SAX2 strong anion exchange	73	67	96	83	22	22	95	91	14	10
Pancreas	IMAC3-Cu WCX	120	60	N/A	N/A	120	60	78^2^	97^2^	9	11
Prostate	IMAC3 copper activated	159	167	98	94	30	30	83	97	9	12
Prostate	C16 hydrophobic interaction	25	31	NR^11^	NR^11^	228	38	95	78	7	13
Prostate	IMAC3-Cu WCX2	30	44	N/A 89^1^	N/A 87^1^	26	62	66 63 85^1^	38 77 85^1^	5 6 3^1^	14

1 Combined performance of peaks from both IMAC-Cu and WCX2 ProteinChip® arrays

2 Bootstrap cross-validation using all patients and controls

3 47 healthy controls, 42 patients with benign breast disease

4 Versus healthy controls and benign disease, respectively

5 Cross-validation versus healthy controls and benign disease, respectively

6 Randomly selected 30 cases and controls

7 Cross-validation using combined performance of peaks from both IMAC-Cu and SAX ProteinChip^®^ arrays

8 Cross-validation using two-thirds of cases and controls as the training set, one third as the test set

9 Results of five independent simulation studies

10 Cross-validation using combined performance of peaks from both IMAC-Cu and WCX2 ProteinChip^®^ arrays

11 Training set results not reported

**Table 2:** Types of chip

Old Designation	Current Chip	Biochemical Action of Surface Chemistry
IMAC3	IMAC30 (with hydrophobic barrier)	Bivalent metals can be attached to the chip. Proteins that bind to these divalent metals (eg, Cu^+2^) are bound by the chip.
WCX2	Same (CM10 mimics WCX2 but does not replace)	This is a weak cation exchange chip. It contains negatively charged (anionic) carboxylate groups that will bind proteins with positively charged areas containing high numbers of lysine, arginine, and/or histidine amino acids.
H4	Same (C16 contains 16 CH_3_)	The chip contains multiple chains, each composed of 16 methylene groups. This very hydrophobic microenvironment binds molecules that are hydrophobic
SAX2	Q10 (with hydrophobic barrier)	Strong anion exchanger which is composed of quartenary ammonium groups that are charged positively. This chip will bind proteins/peptides with regions rich in acidic groups,
NP1 and NP2	NP20	General protein binding surface which binds via serine, threonine or lysine
PS1 and PS2	PS10/PS20	Binds via attachment of capture molecules such as antibodies, binding proteins, etc.
SENDID		Incorporates EAM into chip.

**Table 3:** Potential Organization of Triplicates of Samples on Chips (Spots and Chips Randomly Chosen in Each Group)

Group 1	Group 2	Group 3
Sample 1A, Spot C, Chip 7	Sample 1B, Spot A, Chip 5	Sample 1C, Spot F, Chip 2
Sample 2A, Spot D, Chip 5	Sample 2B, Spot C, Chip 3	Sample 2C, Spot A, Chip 4
Sample 3A, Spot A, Chip 4	Sample 3B, Spot F, Chip 1	Sample 3C, Spot G, Chip 7
.	.	.
.	.	.
.	.	.
Sample NA, Spot D, ChipM	Sample NB, Spot G, Chip M−5	Sample NC, Spot B, ChipM+1
